# An analysis of possible off target effects following CAS9/CRISPR targeted deletions of neuropeptide gene enhancers from the mouse genome

**DOI:** 10.1016/j.npep.2016.11.003

**Published:** 2017-08

**Authors:** Elizabeth Anne Hay, Abdulla Razak Khalaf, Pietro Marini, Andrew Brown, Karyn Heath, Darrin Sheppard, Alasdair MacKenzie

**Affiliations:** School of Medicine, Medical Science and Nutrition, Institute of Medical Sciences, University of Aberdeen, Foresterhill, Aberdeen, AB25 2ZD, Scotland, UK

**Keywords:** Neuropeptide, enhancer, CAS9/CRISPR, Off-target effects, Transgenic mouse, CAS9 mRNA, gRNA, 1-cell mouse embryo, Cytoplasmic microinjection, CRISPR design tool, UCSC browser BLAT, PCR, Sanger sequencing

## Abstract

We have successfully used comparative genomics to identify putative regulatory elements within the human genome that contribute to the tissue specific expression of neuropeptides such as galanin and receptors such as CB1. However, a previous inability to rapidly delete these elements from the mouse genome has prevented optimal assessment of their function in-vivo. This has been solved using CAS9/CRISPR genome editing technology which uses a bacterial endonuclease called CAS9 that, in combination with specifically designed guide RNA (gRNA) molecules, cuts specific regions of the mouse genome. However, reports of “off target” effects, whereby the CAS9 endonuclease is able to cut sites other than those targeted, limits the appeal of this technology. We used cytoplasmic microinjection of gRNA and CAS9 mRNA into 1-cell mouse embryos to rapidly generate enhancer knockout mouse lines. The current study describes our analysis of the genomes of these enhancer knockout lines to detect possible off-target effects. Bioinformatic analysis was used to identify the most likely putative off-target sites and to design PCR primers that would amplify these sequences from genomic DNA of founder enhancer deletion mouse lines. Amplified DNA was then sequenced and blasted against the mouse genome sequence to detect off-target effects. Using this approach we were unable to detect any evidence of off-target effects in the genomes of three founder lines using any of the four gRNAs used in the analysis. This study suggests that the problem of off-target effects in transgenic mice have been exaggerated and that CAS9/CRISPR represents a highly effective and accurate method of deleting putative neuropeptide gene enhancer sequences from the mouse genome.

## 1 Introduction

Neuropeptides and their receptors play a crucial role in maintaining the homeostasis of the human nervous system and in maintaining health (Freimann et al., 2016, Davidson et al., 2011). Neuropeptides such as galanin play critical roles in inflammation (Pinter et al., 2013), mood, appetite and alcohol intake and their mis-expression has been associated with inflammatory pain, depression, alcohol abuse and obesity (Lang et al., 2015). Tightly controlled cell and tissue specific expression of neuropeptides is critical to their function and there is evidence that mis-regulation of neuropeptides and/or their receptors is associated with disease progression (Shanley et al., 2010, Shanley et al., 2011). In order to understand the genomic mechanisms that control the expression of the gene that encodes galanin we have previously used comparative genomics to identify candidate regulatory regions within and around the galanin gene (GAL) locus (Davidson et al., 2011). We were able to identify a 1.5 kilobase (kb) region 42 kb upstream from the human GAL locus that we called GAL5.1. We cloned this sequence and made reporter constructs which were then used to generate reporter mouse lines, using pronuclear microinjection, that expressed the β-galactosidase reporter gene in precisely the same cells that expressed GAL mRNA and galanin peptide (Davidson et al., 2011). Moreover, we were able to report significant differences in the activities of polymorphic variants of GAL5.1 in primary hypothalamic neurones (Davidson et al., 2011). The next logical step in the study of GAL5.1 would have involved its deletion from the mouse genome to permit study of the effects of its deletion on the expression of the GAL locus as well as appetite, mood, the inflammatory response and alcohol intake. However, the expense and time required to delete GAL5.1 from the mouse genome using existing embryonic stem cell targeting based technologies precluded this approach.

The last three years have seen the development of a new technology called CAS9/CRISPR genome editing that promises to revolutionise in-vivo biology (Singh et al., 2014). Briefly, CAS9/CRISPR genome editing relies on the ability of the bacterial CAS9 protein to induce a double strand cut in any region of the genome identified by a guide RNA (gRNA) molecule. The CAS9 protein forms a complex with the gRNA molecule which then ascribes sequence specificity on the CAS9 protein. In this way, any sequence within the genome of any species can be targeted provided that the recognition sequence of the gRNA is next to a short 3 base pair PAM (protospacer adjacent motif = NGG) and the CAS9/gRNA complex can be introduced into the cell. However, reports questioning the specificity of the CAS9-gRNA complex have raised fears of the cutting of “off-target” sites by the CAS9/CRISPR system where CAS9-gRNA complexes are able to cut non-target sites that differ from the actual target site by 1–5 base pairs (Fu et al., 2013, Mali et al., 2013). Although the cutting of these off-target sites are orders of magnitude rarer than “on-target” sites nevertheless this phenomenon has raised concerns of the use of CRISPR/CAS9 technologies (Zhang et al., 2015, Peng et al., 2015) although recent studies have gone some way to addressing these fears (Shen et al., 2014). A number of different approaches have been used to address the generation of off-target effects mostly involving the production of mutant “nickase” versions of the CAS9 enzyme (Ran et al., 2013) or by generation of versions of CAS9 with reduced off-target selection (Slaymaker et al., 2016). Because RNA is rapidly degraded in cells we explored the possibility that injection of CAS9 mRNA and sgRNA into the cytoplasm of one-cell mouse embryos would reduce or alleviate the introduction of “off target effects” in resulting mouse lines. Thus, instead of microinjecting CAS9 and gRNA expressing plasmids into the pronucleus of 1-cell mouse embryos we chose to introduce gRNA and CAS9 mRNA by their injection into the cytoplasm of 1-cell embryos to minimise the exposure of the genome to CAS9 activity (Horii et al., 2014). To test this approach we deleted the GAL5.1 enhancer from the mouse genome using CAS9 mRNA co-injected with two gRNA molecules flanking the GAL5.1 locus and examined the genomes of these founder lines using a combination of PCR, Sanger sequencing and bioinformatics.

## 2 Materials and methods

### 2.1 Generation of gRNA molecules

gRNA molecules were designed to target the most conserved region within the GAL5.1 and ECR1 enhancers using the optimised CRISPR design tool (http://CRISPR.mit.edu/)(Table 1). These guide sequences were used to make T7 templates as described (Harms et al., 2014) and were transcribed to using a Megashortscript T7 in-vitro transcription kit (Ambion) to produce gRNAs as described in the manufacturer's instructions with modifications by Harms et al. (2014).

**Table 1:** Guide sequence selections (PAM sequence in **bold**) and genomic coordinates targeted in mouse genome.

Target region	Guide sequences	Genomic coordinates targeted (GRCm38/mm10)
GAL5.1a	Guide 4: ATGGCTCCCAACAATGCGCCAGG **(AGG)**	chr19:3,441,174–3,441,196
	Guide 8: CGTCAGAGGCCCGTGACTAACGG **(CGG)**	chr19:3,441,324–3,441,349
ECR1	Guide 11: GTCAGAACACTACTGTGTAC **(AGG)**	chr4:33,937,559–33,937,601
	Guide 13: GTTCATTTGCAATGTAGCTT **(AGG)**	chr4:33,937,414–33,937,456

### 2.2 Production of genome edited mice

gRNA molecules were microinjected at a concentration 10 ng/μl each into the cytoplasm of 1-cell C57/BL6 embryos as described (Harms et al., 2014) together with 10 ng/μl CAS9 mRNA (Life Technologies). Surviving two-cell embryos were introduced into host CD1 mothers using oviduct transfer as previously described (Nagy et al., 2003). Once weaned surviving pups ear-clip biopsies were recovered from these animals and stored frozen for further analysis.

### 2.3 DNA extraction

600 μl of 50 mM NaOH was added to each ear-clip biopsy sample and tubes heated at 95 °C for 10 min. After vortexing the solution was neutralised with 50 μl of 1 M Tris HCl (pH 8) and spun at 13,000 rpm for 5 min. The topmost 400 μl was then extracted for subsequent PCR analysis. Correctly deleted loci were detected by PCR using the oligonucleotide pairs shown in Table 2.

**Table 2:** PCR primers used to detect correct targeting events.

Targeted locus	Deletion detection primers
ΔGAL5.1	AGTTAGGGCGCACACATCAA
	CCGTGACTAACGGCTAATGC
ΔECR1	TGTGTGCAGAGAGGGGAGAC
	CTTTAGGAGTGGACAAGGGGTC

### 2.4 Prediction of off-target sites

The CRISPR design tool (http://CRISPR.mit.edu/) also permitted the prediction of likely off-target sites within the rest of the mouse genome based on the sequence of each of the gRNA recognition sequences chosen. We chose putative off-target sequences around the mouse genome that contained the minimal numbers of mis-match regions and which therefore representing the most likely off-target cut sites (Table 3).

**Table 3:** Most likely off-target sites predicted across the mouse genome for guides in Table 1. The “mismatch” column displays how many mismatches (MM) the program detected (2-4MM) and their position (in square brackets) within the predicted off-target sequence when compared to the target sequence.

	Off-target sequence	Mismatches (MM)	Genomic coordinates (GRCm38/mm10)
Guide 4	1.CTGGCTCCCAAAAATGTGCCTGG	3MMs[1:12:17]	chr15:4,531,305–4,531,327
	2.TTTCCTCCCAACAATGAGCCTAG	4MMs[1:3:4:17]	chr15:34,336,397–34,336,419
	3.AAGGCTCACTACAATGTGCCAAG	4MMs[2:8:10:17]	chrX:58,284,910–58,284,932
	4.TTGGAAGCCAACAATGCGCCAAG	4MMs[1:5:6:7]	chr17:27,845,339–27,845,361
Guide 8	1.AGCTAGAGGCCTGTGACTAATAG	4MMs[1:3:4:12]	chr4:36,468,479–36,468,501
	2.AATCAGTGTCCCGTGACTAAAGG	4MMs [1:2:7:9]	chr11:35,124,332–35,124,354
	3.AGGCAGAGGACCGTGAGTAAAGG	4MMs[1:3:10:17]	chr4:109,793,697–109,793,719
	4.GGTAAGTGGCCCATGACTAAGGG	4MMs [1:4:7:13]	chr7:139,558,969–139,558,991
Guide 11	1.ATCTCCATGGAAAAATAAGGCAG	2MMs[1:10]	chr4:7,893,773–7,893,795
	2.CTCCTCATGCAAAAATAAGGAAG	2MMs[4:5]	chr14:98,049,760–98,049,782
	3.CTCTCAATGGAAAAATAAGGGAG	2MMs[6:10]	chr6:46,598,551–46,598,573
	4.GTATCCAGCCAAAAATAAGGTAG	4MMs[1:3:8:9]	chr4:154,290,932–154,290,954
Guide 13	1.GTTTTTTTGCAATGTAGCTTTAG	2MMs[4:5]	chr12:38,313,780–38,313,802
	2.ATTCTTTGGAAATGTAGCTTGAG	4MMs[1:5:8:10]	chr8:49,053,837–49,053,859
	3.TTTCATTTGAAGTGTAGCTTAAG	3MMs[1:10:12]	chr6:5,207,740–5,207,762
	4.CTTCTTTTGGCATGTAGCTTCAG	4MMs[1:5:10:11]	chr6:24,449,864–24,449,886

### 2.5 Identification of flanking regions of putative off-target sites and primer design

Putative off-target sites, as predicted by the CRISPR design tool (http://CRISPR.mit.edu/) were blasted against the mouse genome using the UCSC-genome browser BLAT tool (https://genome.ucsc.edu/cgi-bin/hgBlat). 500 base pair sequences centred on each predicted off-target site were downloaded and entered into the NCBI primer design program (http://www.ncbi.nlm.nih.gov/tools/primer-blast/) to identify optimal primers to aid amplification of PCR products for subsequent sequencing. PCR primers were designed that flanked putative off-target sites at an optimal distance of 100–150 base pairs to give PCR product lengths of between 200–300 base pairs in order to aid in subsequent sequencing (Table 4).

**Table 4:** Primer sets used to amplify predicted off-target sites in the genomes of founder strains of ΔECR1 and ΔGAL5.1 CAS9/CRISPR targeted mice. The annealing temperature of each oligonucleotide and the predicted amplicon length are also shown.

Off-target sequence	Primers (5′-3′)	Annealing temperature (°C)	PCR product size (bp)
**#4/1**	Forward: TCCTACTCTCACAGGGGAGTC	58.3	284
	Reverse: GAATGCTTCTCCCAGGGCAT	63.7	
**#4/2**	Forward: TGGAAGTCCCTCTCCTTGGG	63.8	292
	Reverse: CAGGGTCTCAGGACAACACC	60.6	
**#4/3**	Forward: GGGCACCCTTTCTGTCTGTA	60.1	456
	Reverse: TCTTGCAGGGCTCAGAATGTT	62.2	
**#4/4**	Forward: AAGATCCCTCCTGCTCCGTTC	64.1	232
	Reverse:CACAACAGAGGAAATTATGCGGTT	63.4	
**#8/1**	Forward: TCTTTGATCCAGCAGAGGCT	60.1	235
	Reverse: TCCTGTAACTGCCCCTCAGT	59.7	
**#8/2**	Forward: AGCATTCCTATCCCTCGTCC	60.4	223
	Reverse: AGTTTCACCAGTGGGGATCAG	61.3	
**#8/3**	Forward: GTGCTTCTTGTTGAGCAGCC	61.1	341
	Reverse: CTATCTTGCTGGGTGGAGGC	62.1	
**#8/4**	Forward: TGATCTCCTTCCCGTCCCAT	64.0	283
	Reverse: TGCCCTGTACAACCCCATTC	63.0	
**#11/1**	Forward: CTGGCTATGCTTTTGGTTGATGTT	63.6	305
	Reverse: AAAGATGTTGGGTGTGAATTGAA	60.6	
**#11/2**	Forward: ATGAGGCCAGGAGCCAGATTA	63.2	207
	Reverse: AATTACTTCACCCTCGTGGTCC	61.5	
**#11/3**	Forward: GTCCAGAGAGGAGCATTGGG	62.1	316
	Reverse: AGGCTGCAGTTAGTGTTGTCA	58.6	
**#11/4**	Forward: CCCGGCAAGGCTCAGTAATA	62.4	230
	Reverse: CAGTAGCAAGGGGGACTCAG	59.9	
**#13/1**	Forward:ACCTCTTTCTAGTTCTTCAAATGGA	58.9	254
	Reverse: GTCAGAAAGTCGAACAATCTGTG	58.9	
**#13/2**	Forward: CCAAAGAGGTCAGGCTGTGT	60.3	215
	Reverse: ATCAGAACTTACGCCACCCT	58.7	
**#13/3**	Forward: GAGGCTACTTCCTGTGGCAT	59.3	240
	Reverse: GGAAGGAAGGCCCTAGACAC	60.1	
**#13/4**	Forward: AATGCAGTTCCAGGGAGACC	61.4	267
	Reverse: GTCTGATGAATGCTGCACCC	61.7	

### 2.6 PCR analysis

Test gradient PCR reactions were undertaken on each primer pair (Eurofins) using wild type DNA to optimise their annealing temperatures. Earclip DNA from each founder line was then used as a template to amplify regions of DNA centred on putative off-target sites using a GoTaq polymerase mix according to manufacturer's instructions (Promega). Amplified DNA was then purified using Qiaquick PCR purification kit (Qiagen) and adjusted to 80 ng/μl^− 1^. PCR products were analysed using 1% agarose gel electrophoresis.

### 2.7 Sequencing

Purified PCR products were sequenced by GATC-Biotech sequencing services using each of the PCR primers used for the PCR reaction (See Table 3).

### 2.8 Sequence analysis

**Fig. 1:**
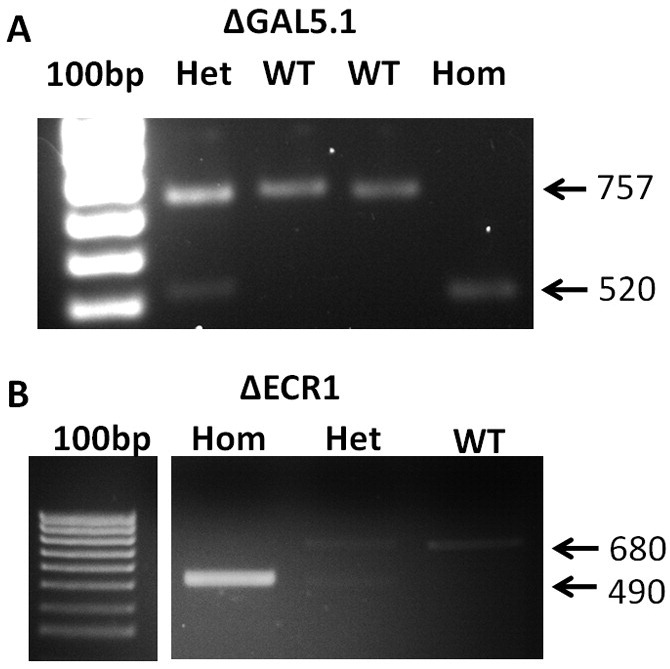
PCR analysis of earclip DNA derived from CRISPR genome edited mice using primer pairs shown in Table 2 demonstrating successful deletion of target loci. Mice were generated using the guide sequences in Table 1 and the change in the sizes of the PCR product reflects successful deletion of the targeted locus using the guide RNAs shown in Table 1. Hom, Homozygous deletion at both loci; Het, Heterozygous deletion at only one locus; WT, wild type loci. 100 bp, 100 base pair ladder marker.

**Fig. 2:**
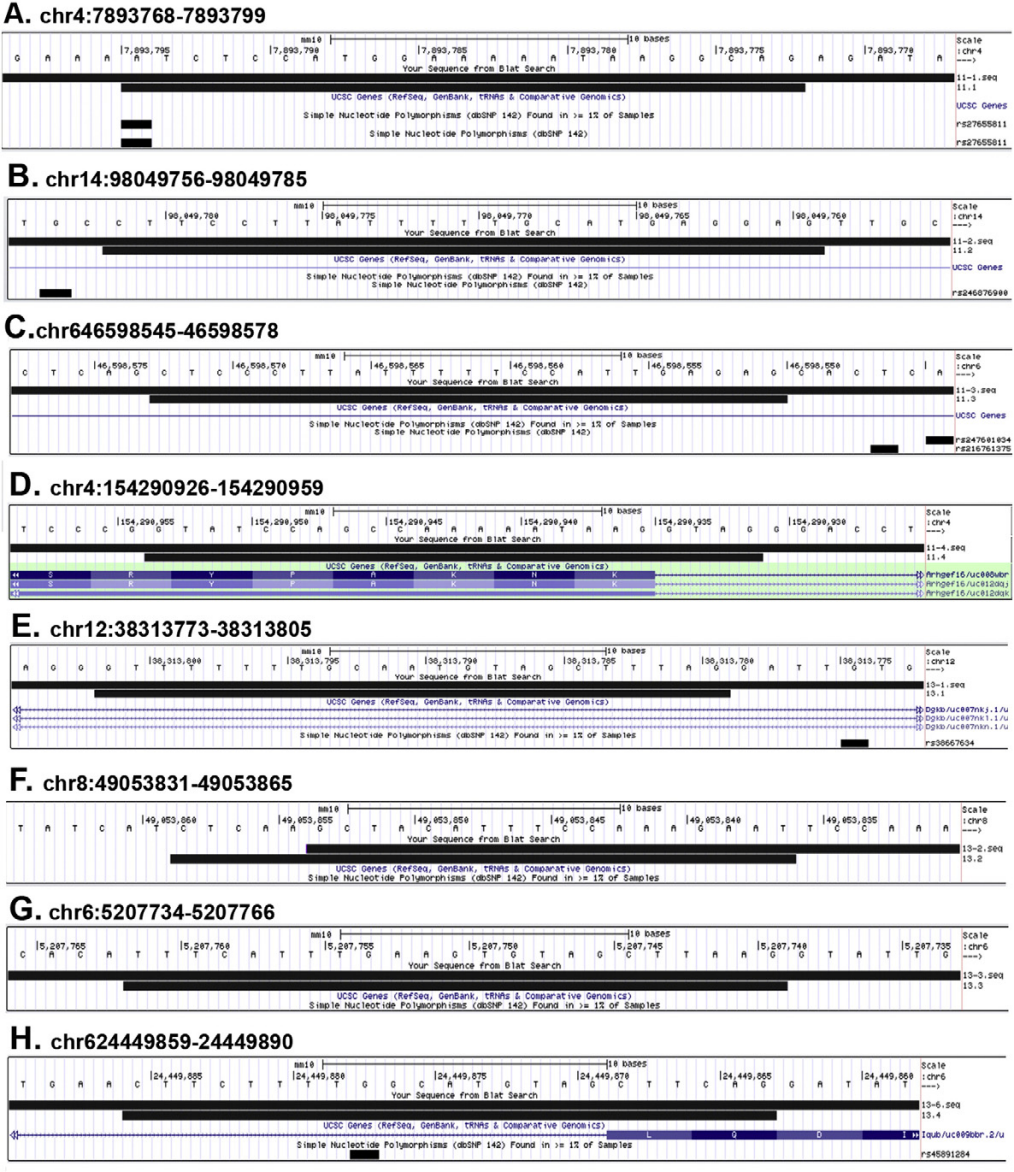
A-M, UCSC genome browser BLAT comparisons (https://genome.ucsc.edu/cgi-bin/hgBlat) of sequencing data (long top black bar) derived from PCR products amplified from earclip DNA of heterozygous (A-F) and homozygous (G-M) ΔGAL5.1 founder lines compared to the sequence of the predicted off-target site (medium length bottom black bar) as predicted by the CRISPR design tool (http://CRISPR.mit.edu/) (Table 2). Differences in sequence are highlighted by the presence of a white letter A, T, C, G or N within either black line. The position of each off-target site along the chromosome is designated numerically above each comparison together with the DNA sequence. Single polynucleotide polymorphism loci are indicated in A, C, F, G, I, J and M as short black bars to highlight possible confounding sequence changes. Exonic sequences are displayed as grey bars in F and M. Scale bars = 10 base pairs.

Sequence information obtained from GATC-Biotech Sequencing services was blasted against the mouse genome using the UCSC-browser BLAST tool (https://genome.ucsc.edu/cgi-bin/hgBlat) (See Fig. 1, Fig. 2).

## 3 Results

### 3.1 Generation of gRNA

gRNA targeting the GAL5.1 and the ECR1 enhancers was generated using the CRISPR designed tool as described above and two guides flanking the most conserved region of both enhancers were chosen (Table 1).

### 3.2 Generation of GAL5.1 and ECR1 targeted mice

Five four-week old female C57/BL6 mice were superovulated and mated to C57/BL6 studs to provide 100–120 one-cell embryos. > 90% survival was obtained after cytoplasmic injection of our gRNA/CAS9mRNA mixture and surviving two-cell embryos were transferred into host female mice. In the case of attempts to delete the GAL5.1 enhancer out of 10 pups that were born we were able to detect two animals that contained the desired deletion for the GAL5.1 enhancer (one heterozygote and one homozygote deletion see Fig. 1A). Crossing of these lines produced Mendelian proportions of heterozygotes and homozygote offspring ruling out the possibility of chimerism in the founder stock. In the case of the ECR1 enhancer we also successfully generated a number of homozygous ECR1 knockout animals( Fig. 1B)). These animals were referred to as ΔGAL5.1 and ΔECR1 respectively. Earclip DNA from a random sample of these founder animals was used in subsequent PCR analysis to detect the possibility of off-target effects.

### 3.3 Amplification of predicted off-target sites

Based on the numbers of mis-matches to the target sequences for each gRNA as calculated by the CRISPR design tool (http://CRISPR.mit.edu/) we chose the four most likely off-target sites predicted for each of the four gRNA molecules using the optimised CRISPR design tool(Table 3). We reasoned that regions of the genome most similar to the target sequence would represent the most likely off-target sequences. We blasted these sequences against the UCSC browser and used 500 base pair genome sequences centred on the predicted off-target sites to design PCR primers. PCR reactions directed to amplify these sites were optimised using gradient PCR and PCR products containing the top four most likely off-target sites, as predicted by the CRISPR design tool, were successfully generated (Table 4). Together with their respective sequencing primers these PCR products were sent to GATC-Biotech sequencing services (https://www.gatc-biotech.com/en/index.html) who were able to provide high quality sequence data for further analysis.

### 3.4 Bioinformatic analysis

**Fig. 3:**
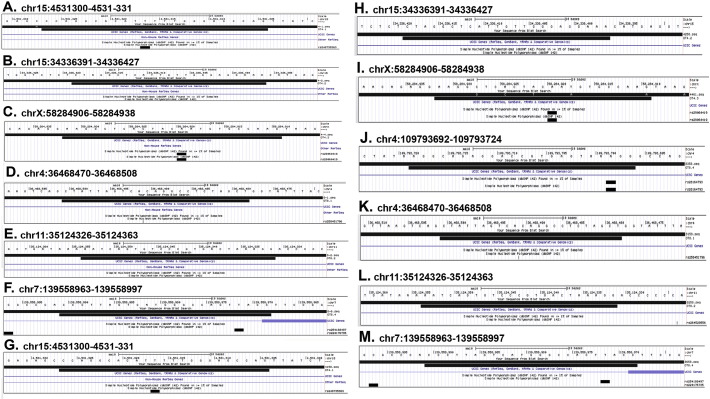
A–H, UCSC genome browser BLAT comparisons (https://genome.ucsc.edu/cgi-bin/hgBlat) of sequencing data (long top black bar) derived from PCR products amplified from earclip DNA of a homozygous ΔECR1 founder mouse compared to the sequence of the predicted off-target site (medium length bottom black bar) as predicted by the CRISPR design tool (http://CRISPR.mit.edu/) **(**Table 2**).** Differences in sequence are highlighted by the presence of a white letter A, T, C, G or N within either black line. The position of each off-target site along the chromosome is designated numerically above each comparison together with the DNA sequence. Single polynucleotide polymorphism loci are indicated in A, B, C, E, and H as short black bars to highlight possible confounding sequence changes. Exonic sequences are displayed as grey bars in D and H. Scale bars = 10 base pairs.

Sequences generated by sequencing of PCR products amplified from earclip DNA from each of our three founder mouse lines were blasted against the mouse genome (GRCm38/mm10) using the Blat tool in the UCSC browser. The results of these blast analyses are shown in Figs. 1 (ΔGAL5.1) and 2 (ΔECR1). Despite the fact that many of the predicted off-target sites contained as few as two mis-matches (Table 2, Guide 11.1 and 11.2 and guide 13.1) none of the sequences amplified from the earclip DNA of any of the three founder lines contained any evidence of CAS9 mediated deletions or base pair changes in any of the four most likely predicted off-target sites (Fig. 2, Fig. 3).

## 4 Discussion

We have used microinjection of gRNA and CAS9 mRNA molecules into the cytoplasms of 1-cell mouse embryos to successfully delete two highly conserved gene enhancer regions (GAL5.1 and ECR1) from the mouse genome. In order to address concerns about the possibility of the CAS9 and gRNA system generating “off-target” changes to the mouse genome we analysed the genomes of three of our founder mouse lines (one heterozygous, one homozygous ΔGAL5.1 and one homozygous ΔECR1) using a combination of predictive bioinformatics to identify possible off-target sites, PCR to allow amplification and isolation of these sequences and Sanger sequencing and bioinformatics analysis to identify off-target effects in these sites. We analysed the effects of CAS9 on the four most likely off-target cut sites of four different gRNAs in three different founder mouse lines (both heterozygous and homozygous ΔGAL5.1 and one ΔECR1; Fig. 1) to test the hypothesis that, if off-target cutting by CAS9/CRISPR was to be an issue in our analyses, that one or more of these predicted off-target sites would show evidence of deletions, insertions or base pair changes characteristic of targeted double strand cuts and subsequent non-homologous end joining repair mechanisms. Using these approaches we were unable to provide any evidence of off-target effects in the genomes of any of the three CAS9/CRISPR enhancer deletion founder mouse lines generated using cytoplasmic injection of gRNA and CAS9 mRNA into 1-cell embryos.

It was reported that up to five mis-matches are tolerated by CAS9 in the gRNA sequence to potentially cut at hundreds of alternative sites across the genome (Fu et al., 2013, Mali et al., 2013). However, it is important to note that, in all of these studies, the off-target effects caused by the CAS9/CRISPR system occurred in the genomes of transformed cell lines transfected with plasmid-DNA constructs constitutively expressing gRNA and CAS9 protein (Fu et al., 2013, Mali et al., 2013, Jamal et al., 2015). It is not surprising that exposing the genomes of rapidly dividing transfected cell lines to high levels of CAS9 and gRNA molecules through multiple generations would increase the incidence of off-target effects in these cells. Indeed, the effects of increased levels of CAS9 activity was noted by Lin et al. (2015) who described that controlling the expression of CAS9 gRNA expressing plasmids produced significantly less off-target effects (Lin et al., 2015). In contrast to observations of off-target effects in cell lines, studies of the use of CAS9/CRISPR in the generation of transgenic mice have reported that off-target effects are far less of an issue than those observed in cell lines (Shen et al., 2014, Chu et al., 2016). Taken together with the results of the current study, that has been unable to detect any evidence of off-target effects, we are confident that, although the chances of off target events are still present, the dangers of off-target site cutting by the CAS9 enzyme when co-injected as mRNA with gRNA are greatly exaggerated and do not constitute any more of a risk than that encountered using ES cell targeting approaches to deleting genome sequences.

From these and other studies it is clear that CRISPR/CAS9 technology for deleting specific sequences from the genome will revolutionise our understanding of the genome and its role in health and disease thanks to the availability of the whole genome sequence of hundreds of vertebrate genomes and the ease and speed of CAS9/CRISPR genome editing. Although CRISPR/CA9 mediated sequence deletion is rapid and highly efficient, the introduction or alteration of sequences within the mouse genome using 1-cell embryos still remains a challenge. This is because these approaches require the co-injection of a DNA “repair template” designed to trigger the homologous end joining repair pathway in the cell to introduce targeted insertions or mutations. There may be several reasons why this approach is less effective that the deletion method described in the current study. The first is that, because of its relative toxicity compared to RNA, DNA decreases the viability of 1-cell embryos following microinjection. Secondly, it is essential that the repair template be injected into the pronucleus of the 1-cell mouse embryo which is trickier than microinjecting into the cytoplasm and results in reduced embryo viability. Thirdly, because of the perceived problem of off-target effects, these repair templates are most often injected with the mRNA of the mutated “nickase” version of CAS9 that only cuts one strand of the DNA target site thus only inducing the homologous repair pathways within the cell. However, the nickase enzyme is nearly an order of magnitude less efficient than the wild type CAS9 protein. One of the most important challenges that must be addressed when attempting to produce subtle mutations using CRISPR technology is that the injection of repair template, that involves the introduction of a glass needle into the pronucleus of the embryo, does not compromise the integrity of the embryo genome and only produces the designed outcome. If this integrity could be assured and the efficiency of homology directed repair increased, then the future for biology and CRISPR genome editing will be extremely bright.

## 5 Conclusions

Despite these problems the use of targeted genome deletions using CAS9/CRISPR technologies is tremendously exciting and promises to revolutionise our understanding of the role of tissue specific enhancers in the cell specific regulation of neuropeptides and their receptors. We have used comparative genomics to rapidly identify highly conserved tissue specific enhancers of genes encoding neuropeptides that often lie at considerable distances from the start sites of these genes. Being able to delete these enhancers from the mouse genome using CAS9/CRISPR technology allows us to span the huge gap between in-vitro analysis of these enhancers (cell lines and transgenic reporter mice) and allows us, for the first time, to understand the role of these enhancers in-vivo. This novel ability will revolutionise our understanding of the regulation of neuropeptides and will permit a greater understanding of the roles of genetic and epigenetic variation in altering neuropeptide gene regulation in health and disease.
